# REDUCING OPIOID OVERDOSE RISK IN A COMMUNITY CARE COORDINATION CLINIC: RICHMOND HEALTH AND WELLNESS PROGRAM

**DOI:** 10.1093/geroni/igz038.2453

**Published:** 2019-11-08

**Authors:** Faika A Zanjani, Marshall Brooks, Leland Waters, Pamela Parsons, Patricia Slattum

**Affiliations:** 1 Virginia Commonwealth, Richmond, Virginia, United States; 2 Virginia Commonwealth University, Richmond, Virginia, United States

## Abstract

Objective Opioid safety is increasingly important in the care of older adults due to higher risk for negative opioid-related outcomes related to higher prevalence of chronic pain, multimorbidity, polypharmacy, and age-associated changes in drug metabolism and elimination. Evidence-based practices for screening and safe opioid use for older adults are needed. Our project aims to develop, implement, and evaluate a care-coordination workflow and interprofessional clinical opioid misuse screening, support, and referral training to support older adult care. Methods Our research occurs in context of the Richmond Health and Wellness Program (RHWP), a community-based interprofessional care coordination initiative, with interprofessional faculty and students providing on-site integrated care to residents in low-income senior housing communities. Curriculum development, and interprofessional clinical faculty, peer support, and health professions student training have been conducted. Results will discuss the findings from the health professions student training. Findings The Opioid Overdose Risk Reduction Curriculum was delivered via Blackboard and in-person during student orientation. Pre (n=66)/Post (n=59) assessment indicated that after the training, there was an increase in knowledge, specifically change from 50% at baseline, 60% recognized Tramadol as an opioid, change from 80% to 97% understanding that MME represents Morphine Milligram Equivalent, and change from 62% to 93% understanding that 50 MME level greatly increases overdose risk. Only 20%, change from 60%, reported not being able to calculate MME. Conclusions Our findings indicate that opioid safety training within community care coordination is feasible. Future works needs to explore the impact on resident health as the workflow is implemented.

